# The Effect of Environmental Conditions on Biofilm Formation of *Burkholderia pseudomallei* Clinical Isolates

**DOI:** 10.1371/journal.pone.0044104

**Published:** 2012-09-06

**Authors:** Nur Siti K. Ramli, Chua Eng Guan, Sheila Nathan, Jamuna Vadivelu

**Affiliations:** 1 Department of Medical Microbiology, Faculty of Medicine, University of Malaya, Kuala Lumpur, Malaysia; 2 School of Biosciences and Biotechnology, Faculty of Science and Technology, Universiti Kebangsaan Malaysia, Bangi, Selangor, Malaysia; The Scripps Research Institute and Sorrento Therapeutics, Inc., United States of America

## Abstract

*Burkholderia pseudomallei*, a Gram-negative saprophytic bacterium, is the causative agent of the potentially fatal melioidosis disease in humans. In this study, environmental parameters including temperature, nutrient content, pH and the presence of glucose were shown to play a role in *in vitro* biofilm formation by 28 *B. pseudomallei* clinical isolates, including four isolates with large colony variants (LCVs) and small colony variants (SCVs) morphotypes. Enhanced biofilm formation was observed when the isolates were tested in LB medium, at 30°C, at pH 7.2, and in the presence of as little as 2 mM glucose respectively. It was also shown that all SVCs displayed significantly greater capacity to form biofilms than the corresponding LCVs when cultured in LB at 37°C. In addition, octanoyl-homoserine lactone (C_8_-HSL), a quorum sensing molecule, was identified by mass spectrometry analysis in bacterial isolates referred to as LCV CTH, LCV VIT, SCV TOM, SCV CTH, 1 and 3, and the presence of other AHL's with higher masses; decanoyl-homoserine lactone (C_10_-HSL) and dodecanoyl-homoserine lactone (C_12_-HSL) were also found in all tested strain in this study. Last but not least, we had successfully acquired two *Bacillus* sp. soil isolates, termed KW and SA respectively, which possessed strong AHLs degradation activity. Biofilm formation of *B. pseudomallei* isolates was significantly decreased after treated with culture supernatants of KW and SA strains, demonstrating that AHLs may play a role in *B. pseudomallei* biofilm formation.

## Introduction

Biofilms can be described broadly as communities of microorganisms that attach to a surface embedded in an extracellular matrix of polymeric substances [1], [2] comprised largely of polysaccharides, and to a lesser extent, proteins and DNA [3]. Biofilms have been demonstrated to be a key player in bacterial pathogenesis as it promotes bacterial survival or spreading within the host, as well as acting as a matrix shield [4], [5] against host defence factors and antimicrobial agents [6]. Regulation of biofilm formation has been linked to quorum sensing (QS) [7] which is a cell-density-dependent communication network that relies on N-acyl-homoserine lactone (AHLs) for the coordination of gene expression [8].

There are various reports on the effect of environmental factors such as oxygen level, pH, temperature, osmolarity used on biofilm formation among different bacterial species such as *Stenotrophomonas maltophilia* [9], *Pseudomonas aeruginosa* [7] and *Hafnia alvei* strains [10]. Another interesting feature is the differentiation of bacteria into large and small colony variants which occur due to environmental stress (increase in metal ion or antibiotic concentration), or in cultures stored over long periods of time [11]. They are also characterised by decreased susceptibility to antibiotic treatment, reduced carbohydrate metabolism, altered virulence factor expression, elevated biofilm formation capacity [12], [13] and their prolonged *in vitro* persistence [14]. SCVs usually appear in cultures of bacterial populations. They have been described in a number of pathogens including *P. aeruginosa* [15], *S. maltophilia* [16], *Salmonella enterica* [17], *Staphylococcus aureus* [8] and *Burkholderia cepacia* [12], and they are frequently isolated from cases of chronic and/or relapsed infections [17].

The pathogenesis of melioidosis, potentially fatal disease in humans a due to *Burkholderia pseudomallei*, remains poorly defined [18], [19]. The disease occurs in a range of many manifestations which include life-threatening sepsis to chronic low-grade infection. Persistence, relapse and recrudescence are also a common feature of this infection despite prolonged antimicrobial therapy [20], particularly among those who acquire diabetes mellitus later on in life. Some of the known virulence factors of *B. pseudomallei* are including exopolysaccharide capsule [21], lipopolysaccharide o antigen [22], type IV pili [23] and type II, III and VI secretion system [24] but these have not been associated with persistence of the infection in chronic melioidosis. It has been postulated that biofilms may play an important role in persistence by the evasion of the host immune response. [25], [26].

Although *B. pseudomallei* biofilms have been well documented in the literature, the objective of this study was to detect and characterise biofilms that were produced by *B. pseudomallei* isolates obtained from different sites of infection, such as wounds, respiratory tract, urine, splenic biopsy, pus and blood, in order to ascertain strain to strain variation. Additionally, the effects of environmental factors such as temperature, growth medium, pH and glucose on biofilm formation among 28 *B. pseudomallei* clinical isolates, including 4 isolates with large colony variant (LCVs) and small colony variant (SCVs) were investigated. The *Caenorhabditis elegans* killing assay was performed to compare the degree of virulence between the LCVs and SCVs following induction of biofilm formation. AHLs production was determined using thin layer chromatography (TLC) and mass spectrometry. Furthermore, in order to ascertain if AHLs play an essential role in its biofilm development, the ability of *Bacillus* sp. soil isolates to quench the AHL molecules was investigated. Confirmation of this quenching ability was performed by the detection of the *aiiA* gene which codes for the AHL lactonase enzyme [27].

## Materials and Methods

### 2.1. Bacteria and growth conditions

All 28 clinical isolates were acquired from University Malaya Medical Centre (UMMC). *B. pseudomallei* isolates were identified by their ability to grow on Ashdown agar (a selective media for *B. pseudomallei*), and by PCR confirmation using *groEL*-specific primer (designated PI 20083144) [28] for genus identification and *mprA*-specific primer (PI 20083144) [28] for species detection. All primers used in this study were synthesised by Nano Life Quest Sdn Bhd, Malaysia. For further confirmation, substrate utilization tests were performed using API20NE test kit according to manufacturer's instructions. Amongst these 28 isolates, four isolates (termed TOM, CTH, VL and OCY) produced two colony morphotypes of large colony variant (LCV) and small colony variant (SCV). Undifferentiated wild type isolates were numbered from 1 to 23. *B. pseudomallei* strain K96243 which is a reference strain has been used as a control strain.

### 2.2. Isolation of small colony variants

Isolates were recovered from nutrient agar (NA) slants and inoculated into 5 ml Luria Bertani (LB) broth and incubated overnight at 37°C with shaking at 150 rpm. A loopful of suspension was inoculated onto nutrient Agar. Colony variants observed were designated as either SCVs or LCVs and were sub-cultured onto fresh NA plates. The constituents of NA included beef extract, peptone and agar. Each colony variants was further biochemically identified as *B. pseudomallei* using the API 20NE kit according to the manufacturer's instructions and by PCR using the *GroEL* and *mprA* primers specific for *B. pseudomallei*.

### 2.3. Quantification of biofilm formation

#### 2.3.1. Biofilm assay in LB

This assay was performed according to the method of Boddey *et al.* [26] with slight modifications. Briefly, 100 µl of LB was dispensed into each well of sterile 96-well polystyrene plates followed by the addition of 1 µl of a bacterial culture grown at 37°C overnight with shaking at 150 rpm. The microtiter plates were incubated at 30°C and 37°C for 18 hr without shaking. Thereafter, 1 µl from each well was transferred into wells of fresh 96-well plates containing 100 µl of fresh LB, in triplicate, and the plates were incubated without shaking for 18 hr at 30°C and 37°C. Following incubation supernatants were carefully removed and the wells were stained with 150 µl of 1% (w/v) crystal violet (Sigma, USA) at room temperature for 30 min. Excess stain was removed and wells were washed twice with 175 µl sterile deionized water, before the addition of 175 µl of dimethyl sulfoxide (DMSO) (Univar, Australia) to solubilise the crystal violet. The plate was then read spectrophotometrically at 570 nm using Varioskan Flash (ThermoScientific, USA). Isolates that formed biofilm had optical density readings of more than 0. Wells containing only the medium were used as background controls. The assay was performed in three independent experiments.

#### 2.3.2. Biofilm assay in MVBM

Biofilm formation in modified Vogel and Bonner medium (MVBM) was determined according to the method of Taweechaisupapong *et al.* [25] with slight modifications. Briefly, a 2% (v/v) inoculum from an overnight culture was inoculated into MVBM and was incubated at 37°C for 18 hr. The culture of each bacterial isolate was then adjusted to an OD_540_ of 0.8–0.9. 200 µl of each bacterial suspension was added in triplicate into sterile 96-well polystyrene plates. The plates were incubated aerobically at 30°C and 37°C for 3 hr to allow bacteria adhesion to the wells. Thereafter, the supernatant in each well was aspirated gently to remove non-adherent bacteria, and was replaced with 200 µl of fresh MVBM. After incubation at 30°C or 37°C for an additional 21 hr, the non-adherent bacteria were again removed and the wells containing adherent bacteria were washed with 200 µl of sterile deionised and fresh MVBM were added. After incubation for an additional 24 hr, supernatants were removed and the wells were washed three times with 200 µl of sterile deionised water. The attached bacteria, representing a two-day biofilm culture, were fixed with 200 µl of 99% (v/v) methanol (Sigma, USA) for 15 min and the plates were dried at room temperature. The wells were stained for 5 min with 200 µl of 2% (w/v) crystal violet and excess stain was removed under running tap water. The plates were air dried and the dye bound to the adherent cells was solubilised with 200 µl of 33% (v/v) glacial acetic acid per well. The OD_620_ of each sample was measured. Isolates that formed biofilm were those with a reading of more than 0. Wells containing only the medium was used as background controls. This assay was performed in three independent experiments.

### 2.4. The effect of glucose and pH on biofilm formation

The effect of glucose on biofilm formation by *B. pseudomallei* was evaluated in LB supplemented with different concentrations of glucose (2 mM, 10 mM, 50 mM, 100 mM, 150 mM, and 200 mM).The effect of pH on biofilm formation was evaluated in LB adjusted to different pH values (5.0, 6.9, 7.2, 8.0, and 9.0).

### 2.5. Twitching motility assay

Twitching motility assay was performed according to the method as described previously by Coil and Anné [29]. Each bacterial isolate was grown in 5 ml LB overnight at 30°C and 37°C after which 20 ul of the culture was spotted onto the surface of a nutrient agar plate and left exposed to air to adsorb onto the surface before replacing the Petri dish lid. The agar plates were then incubated at 30°C and 37°C for 96 hours. The twitching motility zone was measured and recorded.

### 2.6. Killing assay

#### 2.6.1. Nematode strains

The wild type *C. elegans* N2 in this study were obtained from the *C. elegans* Research Facility at Universiti Kebangsaan Malaysia, Bangi. The worms were propagated on nematode growth medium (NG medium) and fed on the normal food source, *E. coli* OP50 [30] at 16°C.

#### 2.6.2. Production of germ line proliferation-deficient (Glp) worms

Wild type N2 were made sterile by an RNAi feeding method utilizing the cdc-25.1 RNAi clone as previously described by Kammath et *al.* [31]. The gene cdc-25.1 encodes a CDC25 phosphatase homolog which affects embryonic viability and is necessary for cell proliferation in the germ line. Briefly, gravid worms were laid on cdc-25.1 RNAi plates for 4 hours and prior to transfer to similar plates for an additional 4 hours of egg laying. Afterwards, gravids were removed and eggs were left to hatch and grow in the presence of cdc-25.1 RNAi to produce sterile Glp worms. One-day old adult hermaphrodite Glp worms reared on the cdc-25.1 RNAi clone lawn were used in the *C. elegans* killing assay experiments.

#### 2.6.3. *C. elegans* killing assay

Briefly, *C. elegans* worms were maintained at 16°C on nematode growth medium (NG medium) agar plates seeded with *E. coli* strain OP50 [30]. The *C. elegans* killing assay was performed as described previously by Tan *et al.* [32]. For standard plate assays, 10 µl of overnight *B. pseudomallei* test isolate culture was spread onto a 3.5-cm-diameter NGM agar plate followed by the addition of 30 L4-stage-growing Glp worms. Worm survival was monitored over time. A worm is dead if it does not respond to touch and its pharyngeal pumping could no longer be observed. For each isolate the assay was run in triplicate plates and in three independent experiments. For each killing assay, nematode survival was calculated by the Kaplan-Meier method, and the survival differences were tested for significance using the log rank test with p value<0.001 considered as statistically significant. *E. coli* strain OP50 served as the negative control.

### 2.7. AHL extraction

All bacterial isolates were cultured in Luria-Bertani (LB) medium. LB medium was buffered with 50 mM 3-[*N*-morpholino] propanesulfonic acid (MOPS) to pH 6.8, to prevent spontaneous degradation of AHLs. Extraction of AHL molecules from *B. pseudomallei* culture supernatant for analytical thin layer chromatography (TLC) and mass spectrometry analysis was prepared according to Shaw *et al.* [33] with minor modifications. In brief, supernatant was collected after centrifuging a 10 ml overnight bacterial culture at 4000× *g* for 20 minutes, after which was extracted twice with equal volume of ethyl acetate acidified with 0.5% (v/v) formic acid (Merck, German). The mixture was shaken vigorously for 30 seconds and the two phases were allowed to separate before carefully aspirating the upper layer containing the AHL molecules into a 50 ml Falcon™ tube. The extract was then filtered and evaporated to dryness. Dried residue was dissolved in 50–100 µl of HPLC-grade ethyl acetate (Emsure, Germany).

To examine the ability of *Bacillus* sp. culture supernatant to degrade AHL molecules produced by *B. pseudomallei*, 10 ml of overnight *B. pseudomallei* culture supernatant was treated with equal volume of *Bacillus* sp. culture supernatant for 4 hours at 28°C and the extraction procedure was carried out as before.

### 2.8. AHL Identification using Thin layer chromatography (TLC) and Mass spectrometry analysis

Extraction of AHL molecules from *B. pseudomallei* culture supernatant, in volumes of 30 µl, were applied to C_18_ reversed- phase TLC plates (20×20 cm aluminium sheet, Merck, Germany) and the chromatograms were developed with methanol/water (60∶40, vol/vol) [33]. After development, the solvent was evaporated, and the dried plates were overlaid with a culture of bioindicator strain prepared as follows. A 30 ml overnight culture of *Chromobacterium violaceum* CV026 was used to inoculate 150 ml of liquidified LB medium containing 1.2% (w/v) agar and the culture was mixed thoroughly before spreading over the surface of the developed plate held in a sterile closed plastic container. The coated plate was incubated overnight at 30°C after the agar solidified. The presence of short chain AHL compounds was indicated as purple spots on agar. To visualise long chain AHL compounds with *N*-acyl side chains C_10_ and above, which antagonise AHL stimulation of violacein synthesis, a modification of this basic assay was employed. In this modified assay, N-hexanoyl-L-homoserine lactone (HHL) (Sigma, USA) was added to the semi-solid agar containing CV026 to a final concentration of 75 nM and the assay procedure was carried out as before [34]. The presence of long chain AHL compounds was defined by the appearance of white halos in a purple background due to inhibition of violacein production. Identification of AHLs was performed using mass spectrometry at the School of Botany, University of Melbourne, Australia.

### 2.9. Quorum quenching study

#### 2.9.1. Soil sample processing and isolation of soil bacilli

Bacteria from soil were investigated for their ability to quench quorum sensing molecules of *B pseudomallei*. Soil samples were collected from two independent locations, with no special permission required as these locations are of outdoor public areas, and then placed in sterile Falcon™ tubes and processed according to the method of Chan *et al.* [35]. All large particles and plant materials were removed from the soil prior to re-suspension of 20 g of soil in 20 ml of sterile distilled water in a sterile Falcon™ tube. The soil suspension was vortexed and placed in a water bath at 100°C for 5 min with gentle shaking, after which the soil suspension was incubated at room temperature for a further 2 hr. The suspension was then serially diluted and spread on LB agar plates. The LB plates were then incubated at 28°C for 16 hr. Single colonies were isolated.

#### 2.9.2. PCR amplification of *aiiA* homologue gene and 16S rDNA

The presence of the *Bacillus aiiA* gene which encodes a protein that is able to cleave bacterial AHL signalling molecules was detected using PCR amplification parameters described by Dong *et al.* [27]. The forward primer 5′-ATG GGA TCC ATG ACA GTA AAG AAG CTT TAT-3′, and reverse primer 5′-GTC GAA TTC CTC AAC AAG ATA CTC CTA ATG-3′ was used for *aiiA* confirmation. The thermal cycling conditions for PCR consisted of initial denaturation at 94°C for 10 min, 35 cycles of 94°C (30 s), 58.35°C (30 s), 72°C (1 min), followed with primer extension at 72°C for 5 min. For *Bacillus* sp., detection of the 16S rDNA gene was performed by PCR amplification using the following primer pairs: forward 5′-AGA GTT TGA TCC TGG CTC AG-3′ and reverse 5′-AAG GAG GTG ATC CAG CC-3′. Each PCR was performed consisting of initial denaturation at 94°C for 5 min, followed by 30 cycles of denaturation (94°C for 30 s), annealing (50.75°C for 30 s) and extension (72°C for 1 min). Final extension was initiated at 72°C for 5 min. For all PCR assays, the negative controls used were sterile distilled water. All the primers used in this study were synthesised by Nano Life Quest Sdn Bhd, Malaysia. PCR products showing expected size as determined by agarose electrophoresis were gel excised, column-cleaned and sequenced for identity confirmation.

#### 2.9.3. AHL Bioassay

For AHL-inactivation test, the method described by Dong et *al.* [27] was employed with slight modifications. Briefly, hexanoyl homoserine lactone (HHL) (Sigma, USA) was added at a final concentration 20 µM in 1.5 ml overnight bacterial culture which was then diluted to an optical density (OD) at 600 nm of 1.1. The reaction mixture was incubated at 28°C with shaking and 50 µl of culture was withdrawn at 0, 3, 6, 9, 12 hr of incubation and added to a central well on an LB agar plate containing a lawn of *C. violaceum* CVO26 strain. Soil bacteria were considered to be capable of inactivating bacterial AHLs when the zone diameter of purple colouration of the CV026 culture surrounding the central well decreased over time. For negative controls, the AHL was incubated with *E. coli* strain DH5α cells or phosphate-buffered saline (PBS, pH 7.2).

#### 2.9.4 Biofilm degradation assay using isolated soil Bacilli

Biofilm formation was determined by the ability of cells to adhere to the wall and to the base of a 96-well polystyrene plate [26]. Briefly, 100 µl of LB was dispensed into each well of sterile 96-well polystyrene plates followed by the addition of 0.5 µl of *Bacillus* sp. culture supernatant and 0.5 µl of overnight *B. pseudomallei* culture in LB broth at 37°C with shaking at 150 rpm. The plates were incubated without shaking at 30°C and 37°C for 18 hr. Thereafter, 1 µl from each well was transferred into triplicate wells of a 96-well plate containing 100 µl of fresh LB and the plates were incubated without shaking for 18 hr at 30°C or 37°C. Biofilm quantification was determined using the methods described above [26].

### 2.10. Statistical analysis

All data obtained from experiments were expressed as mean ± standard error. Statistical significance of the readings was tested with the independent-sample two tailed *t* test based on the SPSS software.

## Results

### 3.1. The effects of LB and MVBM on biofilm formation at different temperatures

**Figure 1:**
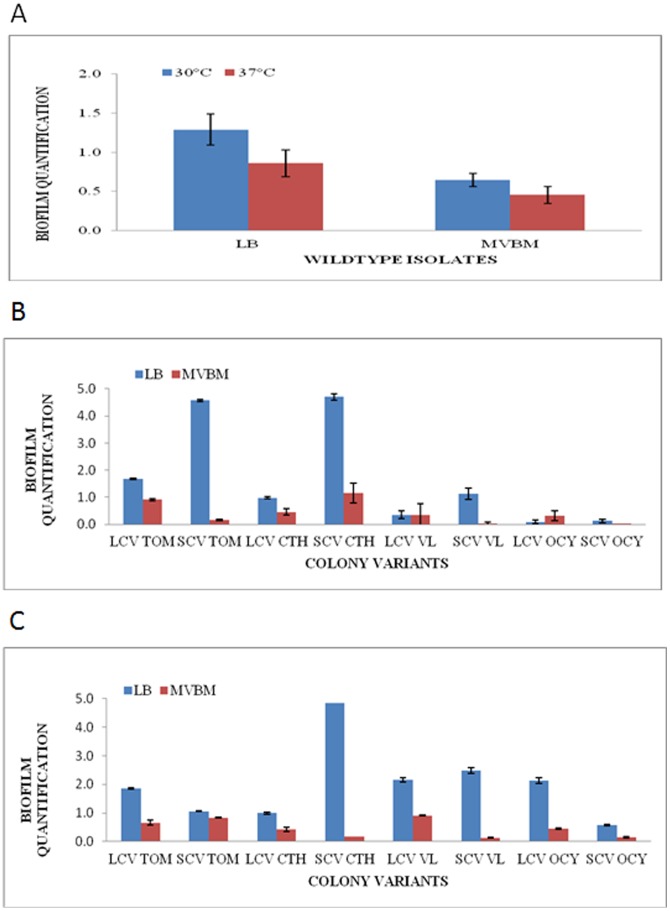
Effect of growth medium on biofilm formation by *B. pseudomallei* wild type and colony morphotypes. Biofilm formation by *B. pseudomallei* (A) wild type isolates at 30°C and 37°C, (B) colony morphotypes at 37°C and (C) colony morphotypes at 30°C, in LB and MVBM. The results in Panel A are reported as the overall mean value for biofilm formation of 24 wild type isolates.

In general, biofilm formation by *B. pseudomallei* occurred at both 30°C and 37°C in both LB and MVBM. However for both medium and temperatures, LB at 30°C facilitated better biofilm production and the amount produced was strain specific. For isolates that did not differentiate into morphotypes, the absorbance values at 30°C for biofilm quantification ranged from 0.338 to 4.685 in LB, and from 0.249 to 1.803 in MVBM. The mean quantities of biofilm formed were 1.264±0.264 in LB and 0.644±0.322 in MVBM. However at 37°C, the absorbance values ranged from 0.075 to 3.7 in LB averaging 0.877±0.183 compared to the biofilm amounts extended from 0 to 2.392 in MVBM averaging 0.453±0.091, suggesting a reduction in biofilm formation. These results demonstrated that the overall mean value of biofilm formation by the 24 wild type isolates was better in LB compared to MVBM, and more biofilms were produced at 30°C than at 37°C (Figure 1A).

Among the four isolates that differentiated into LCV and SVC morphotypes, generally biofilm formation was more extensive in LB compared to MVBM and the amount produced was strain specific. At 30°C, biofilm formation was significantly higher in all morphotypes tested in LB as compared to their counterpart grown in MVBM (*p*<0.05) (Figure 1C). While at 37°C, all morphotypes except LCVs of VL and OCY isolates displayed significantly higher capacities of biofilm formation in LB in comparison with MVBM (*p*<0.05) (Figure 1B). Similarly these results showed that biofilm formation among the morphotypes was better in LB compared to MVBM.

On the other hand, in LB at 37°C, biofilm production was higher in all the SCVs (*p*<0.05) as compared to LCVs, with an absorbance range of 0.124 to 4.704 (Figure 1B). However in LB at 30°C, SCVs of CTH and VL isolates produced increased biofilm with absorbance values of 4.846 and 2.488, respectively, as compared to their isogenic LCV (absorbance readings of between 0.992 and 2.159) (Figure 1C). On the contrary, in MVBM at 30°C, LCVs produced higher amounts of biofilm with the exception of the TOM isolate, and at 37°C, LCV of CTH isolate (OD value 0.453) was found to produce lower amount of biofilms as compared to the SCV morphotypes (OD value 1.152) (Figure 1B).

### 3.2. The effect of pH on biofilm formation

**Figure 2:**
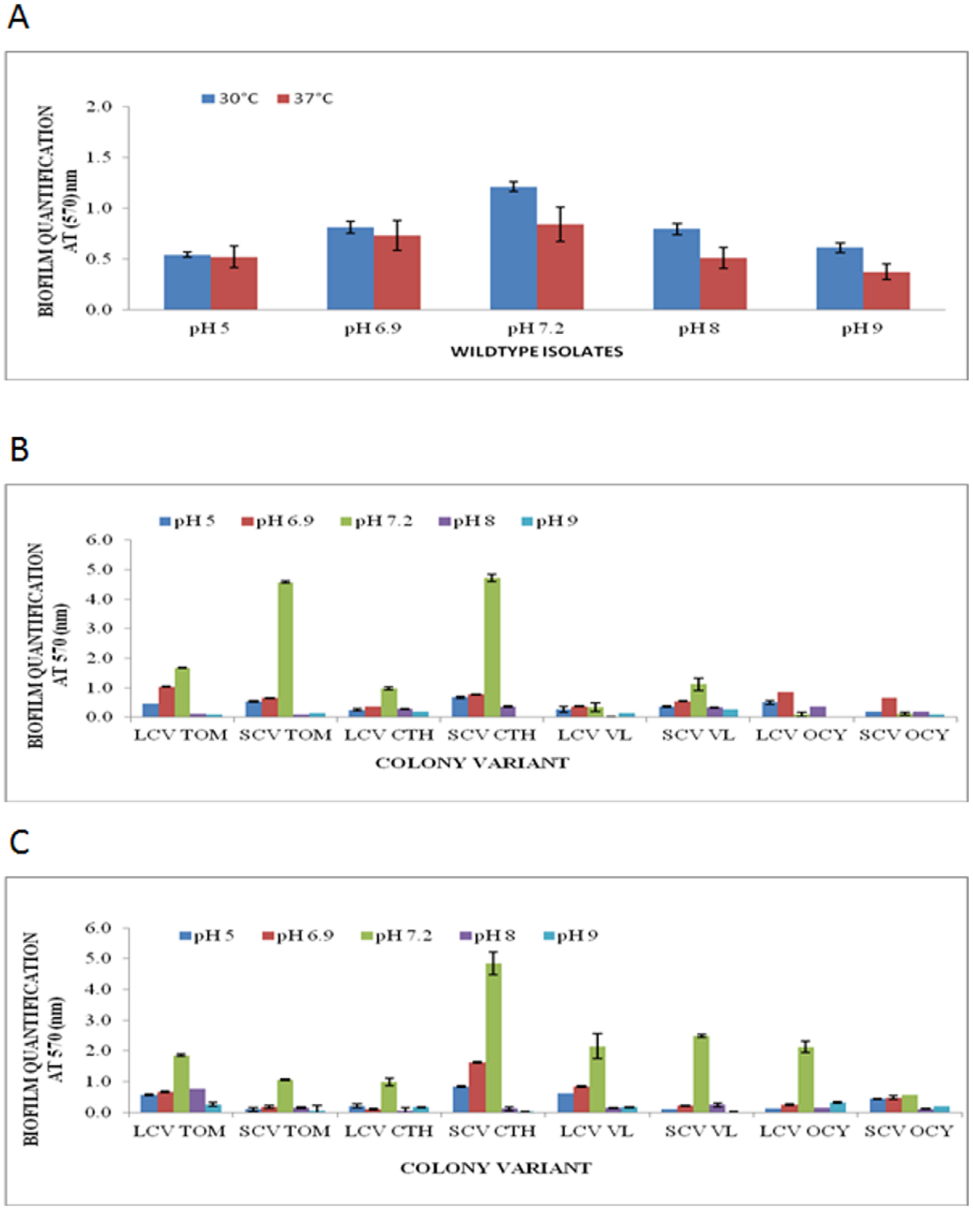
Effect of pH on biofilm formation by *B. pseudomallei* wild type and colony morphotypes. Biofilm formation by *B. pseudomallei* (A) wild type isolates at 30°C and 37°C, (B) colony morphotypes at 37°C and (C) colony morphotypes at 30°C, in LB adjusted to different pH values.

For isolates that did not differentiate into morphotypes grown at 30°C, maximal biofilm formation was achieved at pH 7.2 with an average absorbance value of 1.211±0.194. Biofilm formation was significantly reduced when the medium was more acidic or more basic (Figure 2A). Similar results were obtained at 37°C which the mean biofilm quantity was measured at 0.841±0.164. Among the morphotypes, the optimal pH for biofilm formation was pH 7.2 at 30°C at which all LCVs (OD values of 0.992 to 2.159) and SCVs (OD values of 0.575 to 4.846) morphotypes developed the highest amounts of biofilm (Figure 2C). However at 37°C and pH 7.2, maximal biofilm production was observed only in the LCVs of TOM and CTH isolates, and all SCVs with the exception of the OCY isolate (Figure 2B). The optimal pH for the remaining morphotypes varied. However, the changes in pH during the biofilm studies are possible.

### 3.3. The effect of glucose on biofilm formation

**Figure 3:**
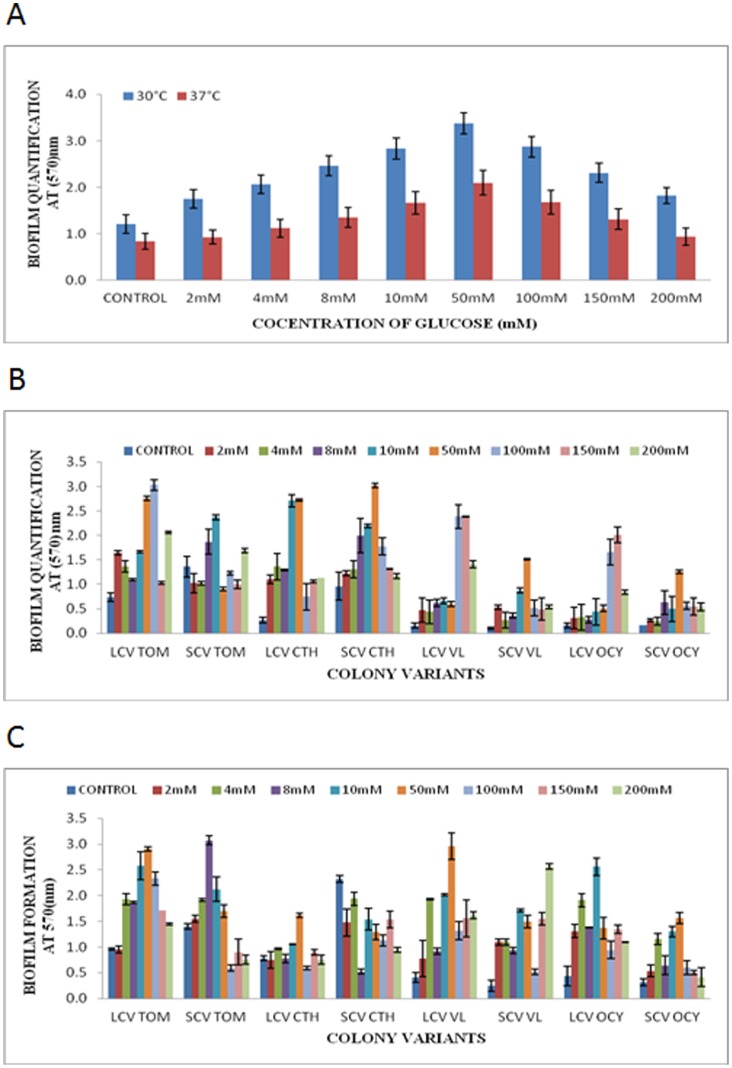
Effect of glucose on biofilm formation by *B. pseudomallei* wild type and colony morphotypes. Biofilm formation by *B. pseudomallei* (A) wild type isolates at 30°C and 37°C, (B) colony morphotypes at 37°C and (C) colony morphotypes at 30°C, in LB supplemented with glucose of different concentrations.

For wild type isolates, it was generally observed that biofilm formation increased significantly (*p*<0.05) in tandem with increasing concentrations of glucose in the medium. Glucose concentration of 50 mM was optimal for biofilm formation at 30°C and 37°C (Figure 3A). Biofilm formation was then gradually diminished with further increasing glucose concentrations. For the LCVs and SCVs, the optimal glucose concentration for biofilm formation varied among each morphotype assayed in this study but generally it was demonstrated that the addition of glucose exerted a positive effect on biofilm establishment in these morphotypes (Figures 3B and 3C). The only exception was the SCV of CTH isolate tested at 30°C, in which the usage of as little as 2 mM glucose had a deleterious effect on its biofilm production.

### 3.4. *C. elegans* killing assay

**Figure 4:**
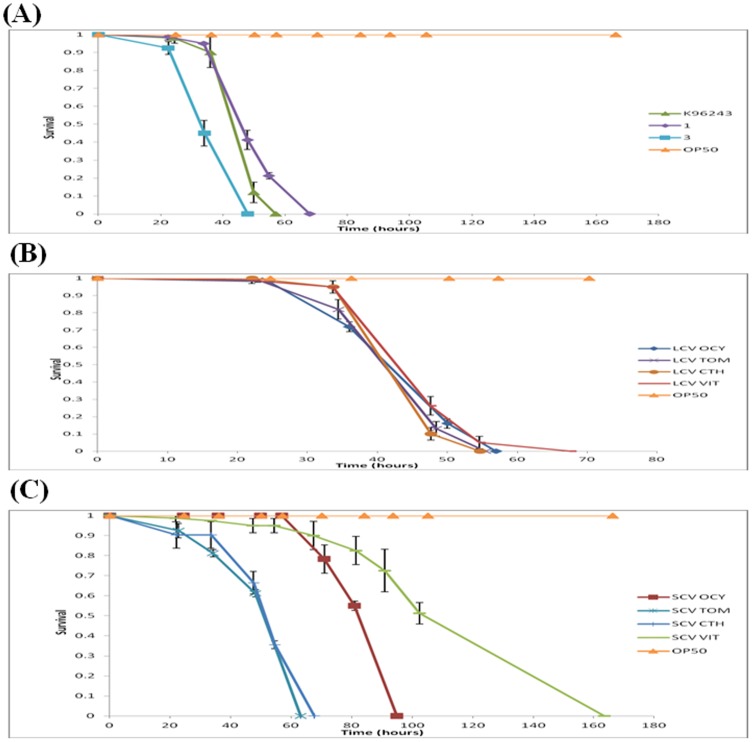
*C. elegans* killing assay. *C. elegans* killing assay by *B. pseudomallei* of wildtype isolates (A), LCVs (B) and SCVs (C).

In this study, we also demonstrated that SCVs were significantly reduced in their *C. elegans* killing activity compared to the LCVs (Figure 4) demonstrating that SCVs were the least virulent in *C. elegans*. Based on the Kaplein Mayer survival statistics, the *C. elegans* killing assay in this study showed that strain 3 was the most virulent, with a mean time to death (TD_mean)_ of 39.437 hours, followed by LCV OCY 46.970 hours, LCV TOM 47.109 hours, LCV CTH 47.804 hours, K96243 49.805 hours, LCV VL 52.08 hours, SCV TOM 53.95 hours, SCV CTH 54.303 hours, strain number 1 55.106 hours, SCV OCY 88.645 hours, with SCV VL being the least virulent (125.96 hours). This study has shown differences in virulence between the wildtype, large colony variants and small colony variants. Generally, it was shown that wildtype and large colony variants are more virulent as compared to small colony variants.

### 3.5. AHL identification

**Figure 5:**
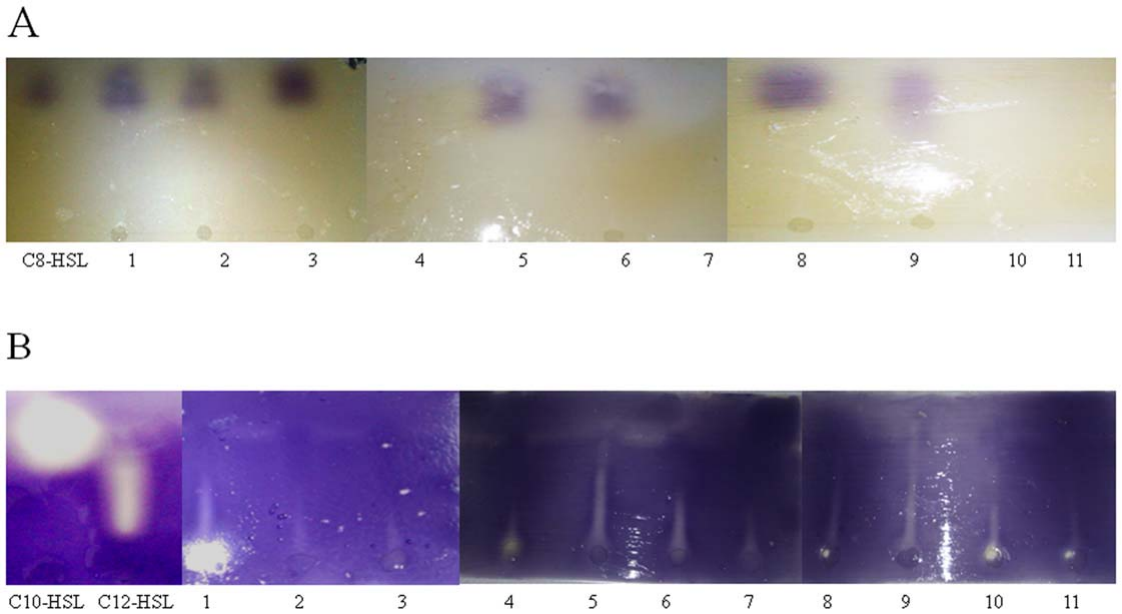
AHL identification of *B. pseudomallei* wild type via analytical TLC assay. TLC analysis of short chain AHL production in wildtype and colony morphotypes of *B. pseudomallei* (A) and long chain AHL production in wildtype and colony morphotypes of *B. pseudomallei* (B). Lane 1: K96342, Lane 2: strain 1, Lane 3: strain 3, Lane 4: LCV TOM, Lane 5: LCV CTH, Lane 6: LCV OCY, Lane 7: LCV VL, Lane 8: SCV TOM, Lane 9: SCV CTH, Lane 10: SCV OCY and Lane 11: SCV VL.

To detect the AHL types produced by *B. pseudomallei* test strains included in this study, thin-layer chromatogram with CV026 overlay technique was employed and has identified C_8_-HSL in all wild type strain, LCV CTH, LCV VL, SCV TOM and SCV CTH, meanwhile, C_10_- HSL were presented in all tested strain except strain number 3 and SCV TOM. All tested strain in this study were produced C_12_- HSL as clearly indicated in Figure 5 (Table 1).

**Table 1:** AHL identification of *B. pseudomallei* isolates using TLC and mass spectrometry analysis.

	Strain	TLC Induction assay (short chain) (C8)	TLC Inhibition assay (Long chain) (C10)	TLC Inhibition assay (Long chain) (C12)	Mass spectrometry (short chain) C_8_- HSL	Mass spectrometry (Potential of Long chain) C_10_- HSL, C_12_- HSL
Wild type	K96243	+	+	+	−	+
	1	+	+	+	+	+
	3	+	−	+	+	+
LCV	TOM	−	+	+	−	+
	CTH	+	+	+	+	+
	VL	+	+	+	+	+
	OCY	−	+	+	−	+
SCV	TOM	+	−	+	+	+
	CTH	+	+	+	+	+
	VL	−	+	+	−	+
	OCY	−	+	+	−	+

Mass spectrometry was employed to re-examine AHL production in 3 selected wild type isolates (K96243, 1 and 3) and in all colony variants. Results for quantitation indicated the presence of of octanoyl-homoserine lactone (C_8_-HSL) molecule in LCV CTH, LCV VL, SCV TOM, SCV CTH, 1 and 3 at 5.4 to 44.4 µM. In addition, results indicated the presence of other AHL's with higher masses (longer chain) (Table 1) were seen in all culture supernatant from wild type and colony morphotypes of *B. pseudomallei* in this study contained N-decanoyl-homoserine lactone (C_10_-HSL) with signature peak 256 and N-dodecanoyl-homoserine lactone (C_12_-HSL) with signature peak 284.

### 3.6. Quorum quenching study

#### 3.6.1. Isolation of soil bacteria and detection of *aiiA* homologue gene

A total of 10 bacterial isolates were screened for the presence of an *aiiA* homologue gene and 2 isolates with an *aiiA* homologue gene amplified by PCR were observed. The expected amplicon size was approximately 800 bp. PCR amplification of the 16S rDNA, expected amplicon size of approximately 1.5 kb, confirmed that these two isolates belonged to the genus *Bacillus*. PCR amplification using *aiiA*-specific primers was therefore conducted and PCR products were successfully acquired.

#### 3.6.2. AHL bioassay

Both soil-derived *Bacillus* sp. isolates, termed KW and SA respectively, which exhibited strong AHL inactivating activity, were tested in AHL inactivation assay to confirm their quorum quenching activity. As anticipated, BLAST analysis of both sequenced genes from SA and KW isolates respectively returned their identity as *aiiA* homologue (JQ844904). Both SA and KW were identified as *Bacillus* sp. based on 16S rDNA gene sequence and BLAST analysis (JQ839268).

**Figure 6:**
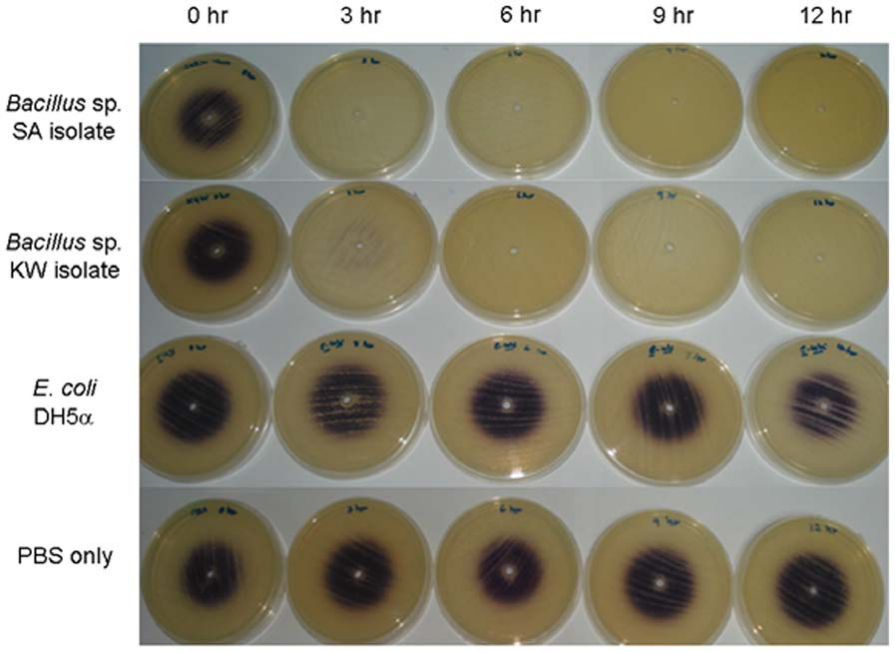
AHL bioassay. The results showed the quorum quenching activity of KW and SA isolates by degrading HHL (disappearance of purple pigmentation) over time. *E. coli* strain DH5α and PBS were included as negative controls.

Purple-coloured zone diameters indicative of HHL presence were recorded in Table 1 and a representative result was shown in Figure 6. All HHL was degraded after incubation with SA isolate for 3 hours, indicating rapid AHL degradation. Similar but less pronounced effect was observed in KW isolate, in which total HHL breakdown occurred after 6 hours incubation. *E. coli* strain DH5α and PBS were included as negative controls, no apparent AHL degradation was observed. SA and KW isolates were further examine using TLC whether can inactivate any of the AHL compounds found in *B. pseudomallei*.

#### 3.6.3. TLC analysis after treatment with KW and SA isolates

**Figure 7:**
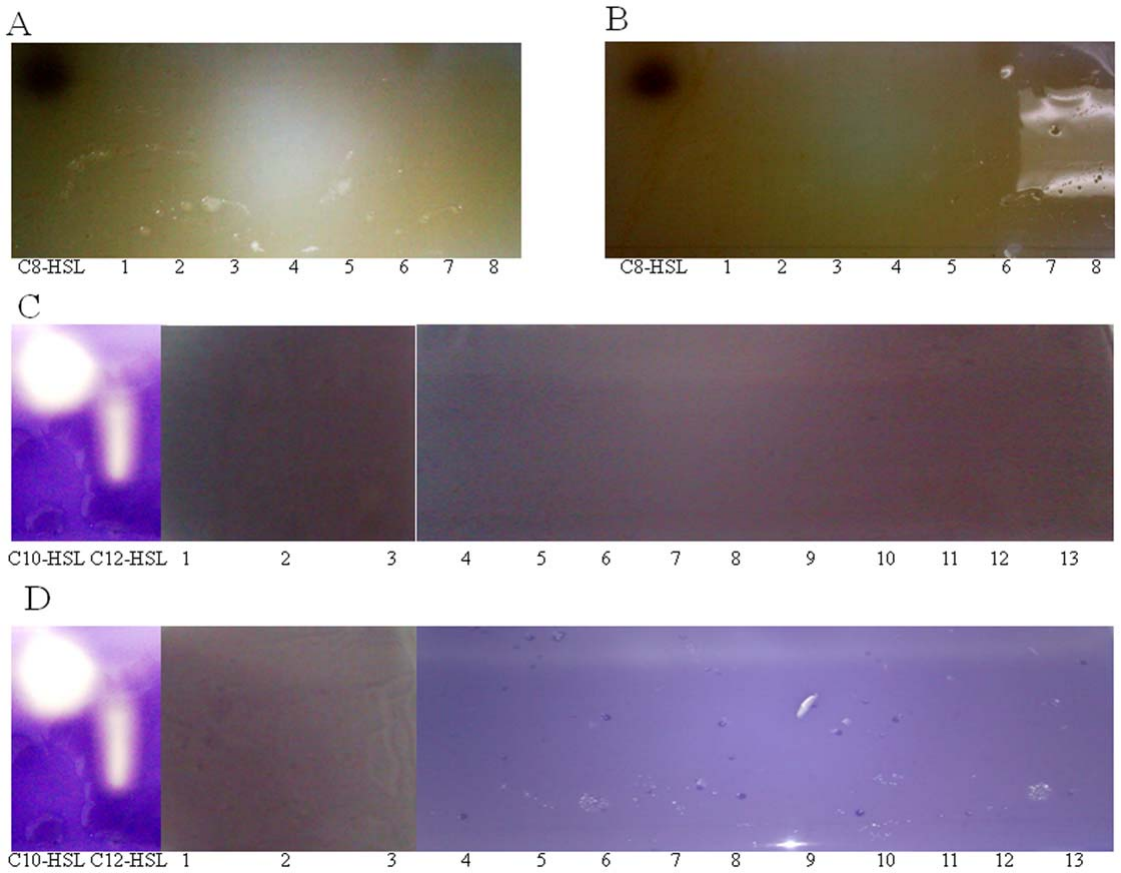
*B. pseudomallei* AHLs treatment with *Bacillus* sp. culture supernatants. Detection of AHL molecules in *B. pseudomallei* bioactive compound extracts prepared after treatment with *Bacillus* sp. culture supernatants. A and C, *Bacillus* sp. KW culture supernatant treated; B and D, *Bacillus* sp. SA culture supernatant treated. Panel A and B: Lane 1: K96342, Lane 2: strain 1, Lane 3: strain 3, Lane 4: LCV CTH, Lane 5: LCV VL, Lane 6: SCV TOM and Lane 7: SCV CTH. Panel C and D: Lane 1: K96342, Lane 2: strain 1, Lane 3: strain 3, Lane 4: LCV TOM, Lane 5: LCV CTH, Lane 6: LCV OCY, Lane 7: LCV VL, Lane 8: SCV TOM, Lane 9: SCV CTH, Lane 10: SCV OCY and Lane 11: SCV VL.

**Figure 8:**
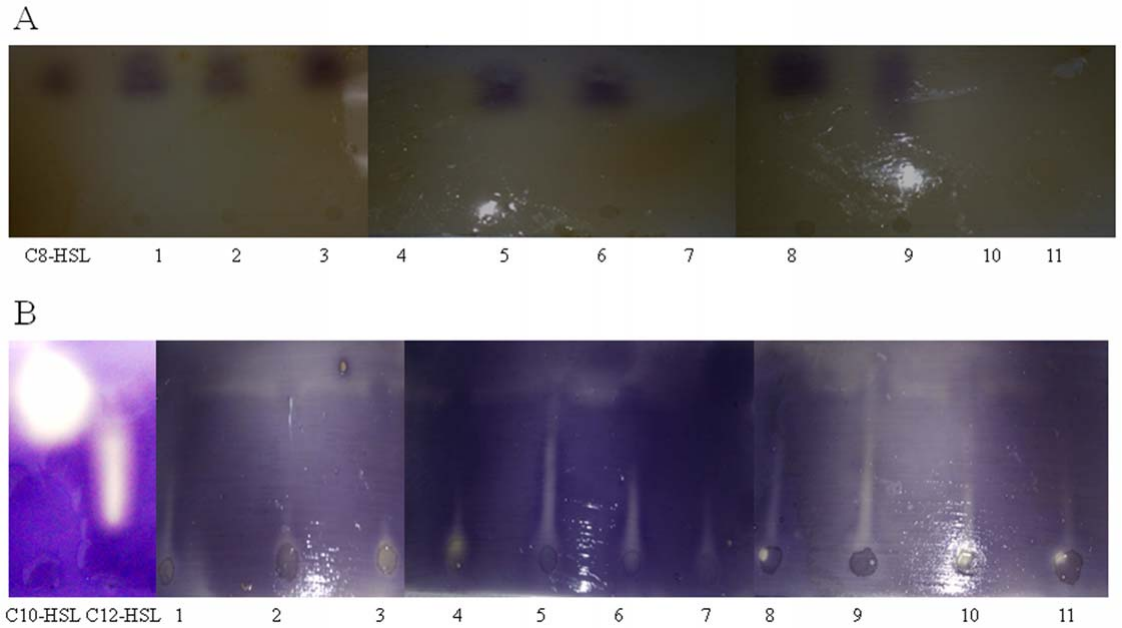
*B. pseudomallei* AHLs treatment with *E. coli* culture supernatants. Detection of AHL molecules in *B. pseudomallei* bioactive compound extracts prepared after treatment with *E.coli* culture supernatants for short chain AHL production in wildtype and colony morphotypes of *B. pseudomallei* (A) and long chain AHL production in wildtype and colony morphotypes of *B. pseudomallei* (B). Lane 1: K96342, Lane 2: strain 1, Lane 3: strain 3, Lane 4: LCV TOM, Lane 5: LCV CTH, Lane 6: LCV OCY, Lane 7: LCV VL, Lane 8: SCV TOM, Lane 9: SCV CTH, Lane 10: SCV OCY and Lane 11: SCV VL.

Once the presence of AHL compounds in *B. pseudomallei* culture supernatant extracts has been confirmed as shown above, we then examined the use of *Bacillus* sp. SA and KW culture supernatants to inactivate AHL compounds generated by *B. pseudomallei* test strains. Results attained via analytical TLC assay demonstrated that C_8_-HSL, C_10_-HSL and C_12_-HSL detected earlier in the tested strain extracts disappeared (Figure 7) after initial treatment with *Bacillus* sp. SA and KW culture supernatants respectively prior to extraction, indicating that both SA and KW secrete an AHL-inactivating enzyme which is capable of degrading QS molecules produced by *B. pseudomallei*. *E. coli* DH5α culture supernatant served as a negative control in this experiment (Figure 8), where no loss of *B. pseudomallei* AHL compounds was observed after 4 hours treatment.

#### 3.6.4. Biofilm quenching assay using soil *Bacilli*

**Figure 9:**
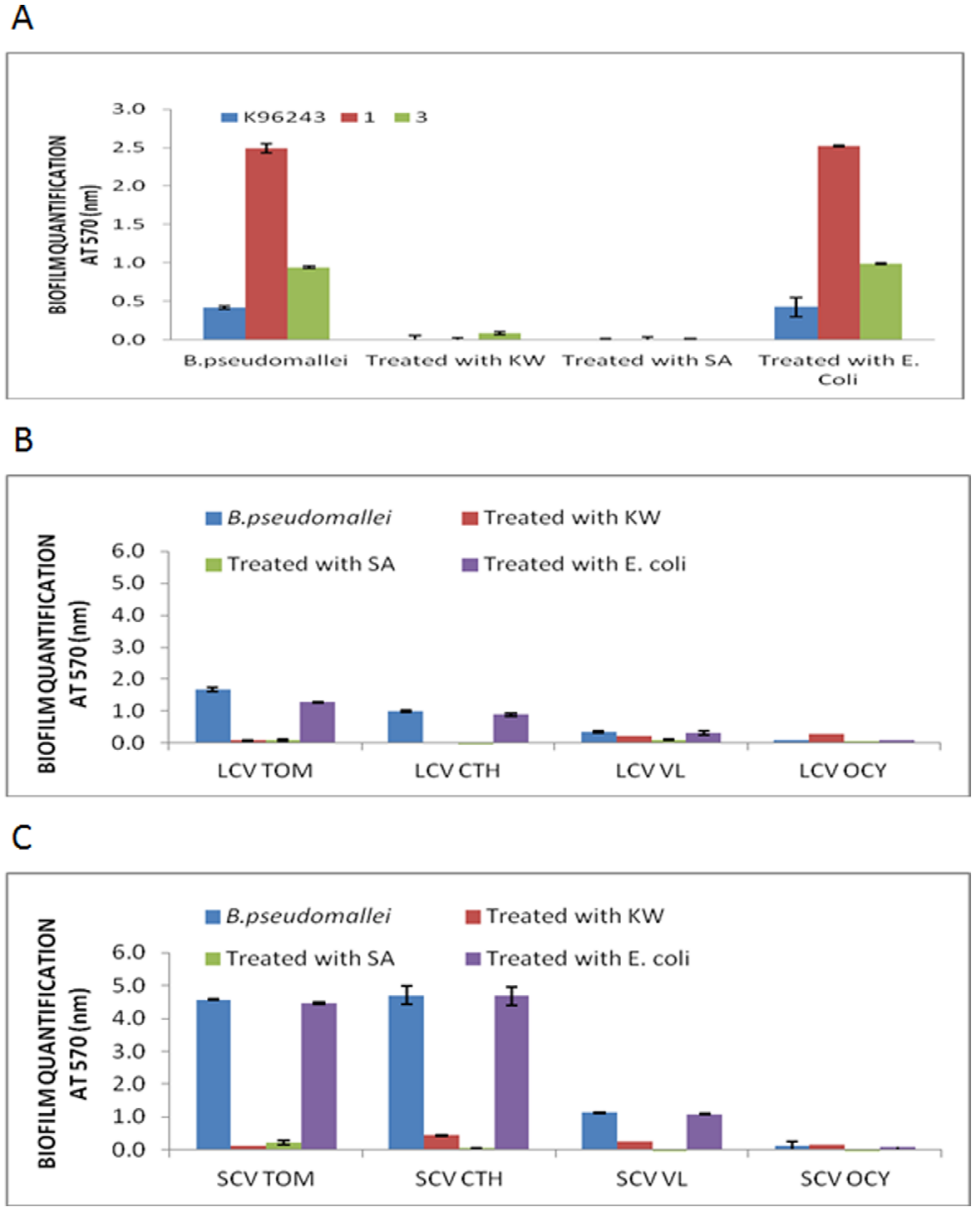
Biofilm quenching assay at 37°C. Inhibition of biofilm formation in *B. pseudomallei* (A) wild type isolates, (B) large colony morphotypes and (C) small colony morphotypes at 37°C in the presence of culture supernatants of quorum quenching *Bacillus* sp. isolates.

**Figure 10:**
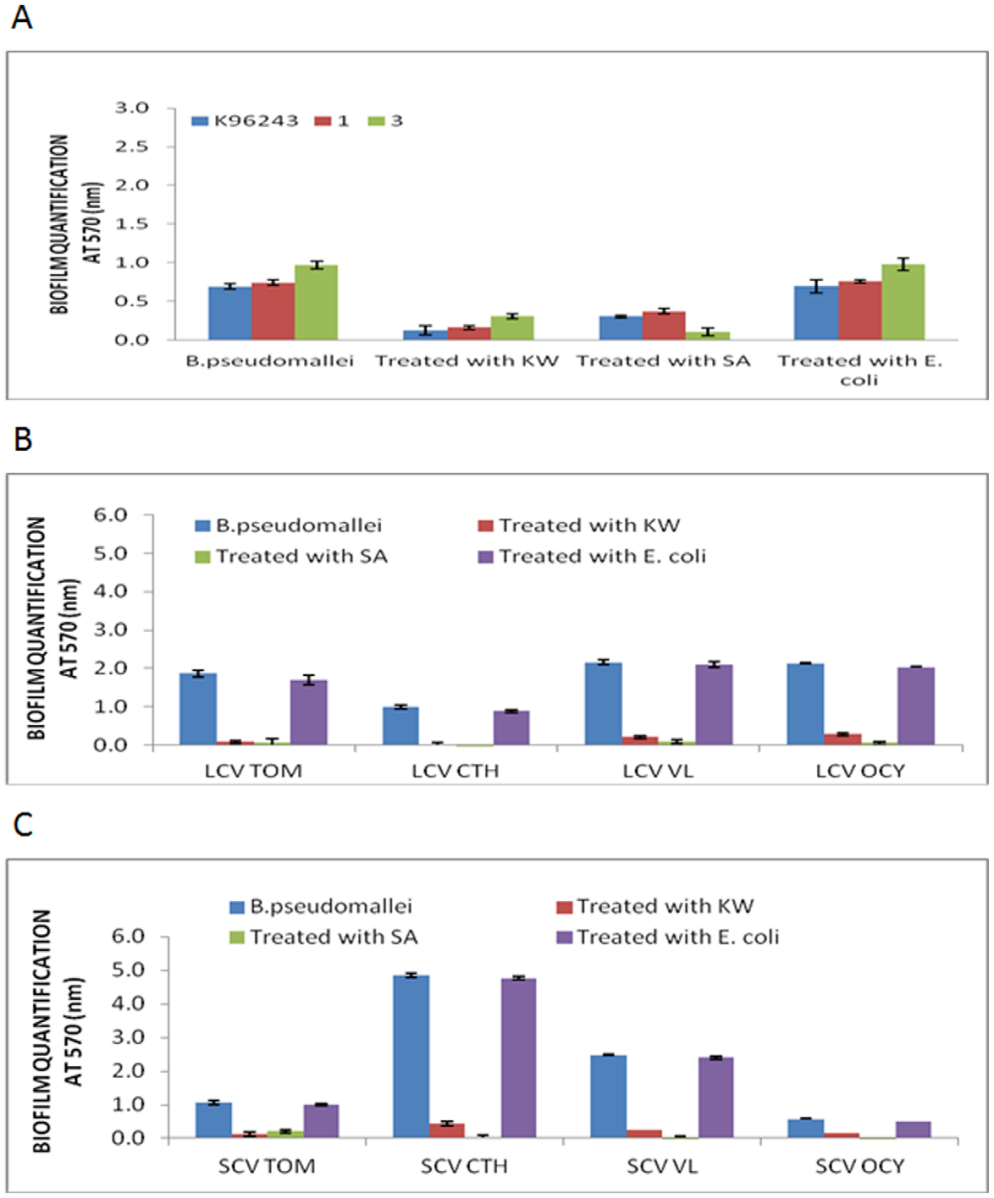
Biofilm quenching assay at 30°C. Inhibition of biofilm formation in *B. pseudomallei* (A) wild type isolates, (B) large colony morphotypes and (C) small colony morphotypes at 30°C in the presence of culture supernatants of quorum quenching *Bacillus* sp. isolates.

It was generally observed that the biofilm formation of wild type isolates and colony morphotypes were significantly reduced (*p*<0.05) at both temperatures 30°C and 37°C after treatment with bacterial supernatants harvested from *Bacillus* sp. KW and SA isolates (Figures 9 and 10). For wild type isolates, biofilm formation at 30°C was significantly reduced from an absorbance range between 0.689 and 0.968 to range of absorbance values of 0.104 to 0.369 after treated with *Bacillus* sp. KW and SA isolates. At 37°C, the OD values for biofilm quantification ranged between 0.414 and 2.491 before treated with *Bacillus* sp. KW and SA isolates were reduced significantly with an absorbance range of 0 to 0.085.

Among the four isolates that differentiated into LCVs and SCVs morphotypes, at 30°C, OD values extended from 0.575 to 4.846 were decreased significantly to an absorbance range of 0 to 0.439 after treated with *Bacillus* sp. KW and SA isolates. At 37°C, biofilm quantification was decreased with range of OD values between 0.093 and 4.704 to range of biofilm readings between 0 and 0.440. Taken together, it was shown that *Bacillus* sp. KW and SA isolates exhibited quorum quenching activity which can impair *B. pseudomallei* biofilm growth possibly by degradation of AHL molecules that may play a role in biofilm formation.

## Discussion

The presence and development of biofilms have now been reported increasingly to play a major role in pathogenesis of disease as it aids bacterial survival within the host shielded in a polysaccharide matrix [4], [5]. Various factors which include environmental factors, types of sugars available in the media, temperature and types of AHL molecules which vary for the different genus of bacteria, have been suggested to play a role in the development and survival of these biofilms in the host.

In this study it was demonstrated that the combination of LB medium supplemented with 50 mM glucose at pH 7.2 and growth temperature at 30°C provides the ideal conditions for biofilm formation in wild type isolates. Our data also showed that *B. pseudomallei* cultured in LB produced more biofilm as compared to culture in MVBM. The constituents of LB included tryptone, yeast extract and sodium chloride, in which both tryptone and yeast extract provide a wide range of peptides and amino acids, vitamins, nitrogen and carbon source which are essential for bacterial growth. MVBM, in contrast, is a chemically-defined high mineral content medium containing MgSO_4_.7H_2_O, anhydrous citric acid, NaNH_4_HPO_4_, K_2_HPO_4_, CaCl_2_.2H_2_O and D-gluconate with a final pH of 7.2 and has been reported in studies on biofilm production of *B. pseudomallei* [25]. In light of the data above, peptides or amino acids may be more favourable essential elements that encourage the development of larger amounts of biofilm. This is an important finding as in the host, bacteria are exposed to large amounts of proteins and peptides that are present in the tissues and body fluids. Therefore it can be envisioned that one of the factors contributing to persistence of *B. pseudomallei* is the formation of biofilms in the host cells, organs and tracts.

Boddey *et al.* [26], reported that extensive biofilm formation of *B. pseudomallei* was achieved at 27°C. Their study demonstrated that although *B. pseudomallei* 08 formed biofilms when cultured at 37°C, the extent of biofilm formation increased when incubated at 27°C, which is the temperature of soil, being the habitat of the bacterium. *B. pseudomallei* infections are acquired from soil and/or water [36], they suggest that the transmission of microcolonies of *B. pseudomallei* formed in the soil may contribute to role in the success of the bacterium to establish an infection in the host. The microcolonies would also enable the bacteria to evade the host's immune response. Our data was also in agreement to that of Boddey *et al.* [26], as the *B. pseudomallei* wild type isolates in our study produced more biofilms at 30°C than at 37°C.

The optimal pH for *B. pseudomallei* biofilm formation was pH 7.2, (pH of human lung is 7.4) suggesting that infection of the human lung provides an ideal niche for colonisation in the host. The presence of as little as 2 mM glucose was found to exert a positive effect on biofilm establishment in *B. pseudomallei* with maximal biofilm formation at 50 mM glucose concentration. This may provide clues as to why patients with diabetes mellitus succumb to more chronic and relapsing infections as compared to non-diabetics [37].

Among colony variants, in general, SCVs were found to produce larger amounts of biofilms at 37°C in LB medium as compared to LCVs. This finding is supported by similar findings among SCVs of *P. aeruginosa* as compared to wild type at 37°C [15]. The role of SCVs, constitute a slow-growing subpopulation of bacteria that have been implicated in persistent and relapsing infections. Therefore it is interesting to understand if SCVs of bacteria would be more successful in establishing an infection and at the same time demonstrate higher degree of virulence. In contrast in the *C. elegans* model it was found that SCVs demonstrated significantly reduced killing activity as compared to the LCVs indicating that SCVs were less invasive. Therefore although the SCVs may remain persistent extracellulary in biofilms, they remain inert as compared to LCVs as they are shielded in a matrix. This may therefore contribute to their reduced virulence as compared to LCVs, although they are able to persist and play a role in chronic or relapsing infections.

Regulation of biofilm formation is achieved by QS signal molecules known as N-acyl-homoserine lactones (AHLs) [7]. In this study TLC demonstrated that a majority of strains produce octanoyl-homoserine lactone, C_8_-HSL, decanoyl-homoserine lactone C_10_-HSL and dodecanoyl-homoserine lactone, C_12_-HSL suggesting that these AHL molecules are ubiquitous in strains of *B. pseudomallei*. AHLs found among all our strains included. dodecanoyl-homoserine lactone, C_12_-HSL. The octanoyl-homoserine lactone, C_8_-HSL was produced by all wild type strains and LCVs of CTH VL and SCVs of TOM and CTH and the decanoyl-homoserine lactone, C_10_-HSL was produced by all tested strain except strain number 3 and SCV TOM. Mass spectrometry analysis identified C_8_-HSL in LCV CTH, LCV VL, SCV TOM, SCV CTH, 1 and 3 and other AHL's with higher masses (longer chain) including C_10_-HSL and C_12_-HSL in all wild type and colony variants in this study. However differences observed between the TLC results and mass spectrometry may be attributed to the low amounts of AHL molecules that are below the detection threshold of *C. violaceum* CV026 biosensor strain [38] in the TLC assay.

Quorum quenching enzymes have been demonstrated to play a key role in counteracting microbial quorum sensing signalling. In this study, *Bacillus* sp. soil isolates demonstrated high quorum quenching activity thus inhibiting biofilm formation in *B. pseudomallei* via AHLs degradation. More importantly, when *B. pseudomallei* culture supernatants were treated respectively with *Bacillus* sp. bacterial supernatants, the AHLs could not be detected demonstrating that the AHL molecules had been degraded. In order to confirm the presence of broad-spectrum AHL-inactivating lactonase enzyme by both *Bacillus* sp., SA and KW, presence of the *aiiA* gene confirmed that the two *Bacillus* sp. were capable of degrading the AHL molecules of our collection of *B. pseudomallei* strains.

## Conclusion

This study demonstrates that the regulation of biofilm formation in *B. pseudomallei* is complex and is influenced in a strain-specific manner by external conditions such as temperature, pH, glucose content and growth medium used. The divergent biofilm responses suggest that *B. pseudomallei* clinical isolates have the potential to form biofilm and the capacity of individual isolates to form biofilm and eventually to cause disease may also be influenced by host factors and environmental conditions at the site of infection. To date the emergence of bacterial antibiotic resistance remains a critical issue due to inappropriate and irrational use of antimicrobial medicines worldwide. Medical researchers are in a dire race against time to develop novel antibacterial approach to ward off deadly bacterial infections, including *B. pseudomallei* that is intrinsically resistant to multiple antibiotics. In recent years, researchers have identified quorum sensing as a novel target for antimicrobial therapy, given the numerous bacteria that employ quorum sensing in the control of virulence. It is therefore of interest that AHL-lactonases of *Bacillus* sp. could offer a protective role against *B. pseudomallei* infection and requires further investigation.
